# Myocardial iron quantification using modified Look-Locker inversion recovery (MOLLI) T1 mapping at 3 Tesla

**DOI:** 10.1186/1532-429X-15-S1-W8

**Published:** 2013-01-30

**Authors:** GC Camargo, T Rothstein, FP Junqueira, E Fernandes, RL Lima, A Greiser, R Strecker, JA Lima, SS Xavier, I Gottlieb

**Affiliations:** 1 CDPI - Clínica de Diagnóstico por Imagem, Rio de Janeiro, Brazil; 2 Medicine/Cardiology, Johns Hopkins University, Baltimore, MD, USA; 3 Cardiology, Hospital Universitário Clementino Fraga Filho - UFRJ, Rio de Janeiro, RJ, Brazil; 4 Siemens Healthcare, Erlangen, Germany; 5 Siemens LTDA, Sao Paulo, SP, Brazil

## Background

Quantification of myocardial iron overload is critical for the management of patients with hemochromatosis. The effects of excess iron on T1 and T2* relaxation times correlate directly with tissue iron concentration. T2* became the clinical standard at 1.5T as it can be easily obtained in a fast one breath-hold ECG gated multi-echo GRE sequence. At 3T, however, T2* quantification can be limited by pronounced susceptibility artifacts and signal sampling restraints due to shorter T2* times at higher iron concentrations . Since myocardial T1 time is up to thirty times longer than T2*, it can be quantified with short echo-time inversion-recovery sequences even at high iron concentrations, and is less sensitive to susceptibility artifacts. We aimed to validate a recently developed modified Look-Locker inversion recovery (MOLLI) sequence to quantify myocardial T1 in healthy controls and patients with iron overload at 3T, comparing to standard GRE based multi-echo T2* times at 1.5T.

## Methods

**Figure 1:**
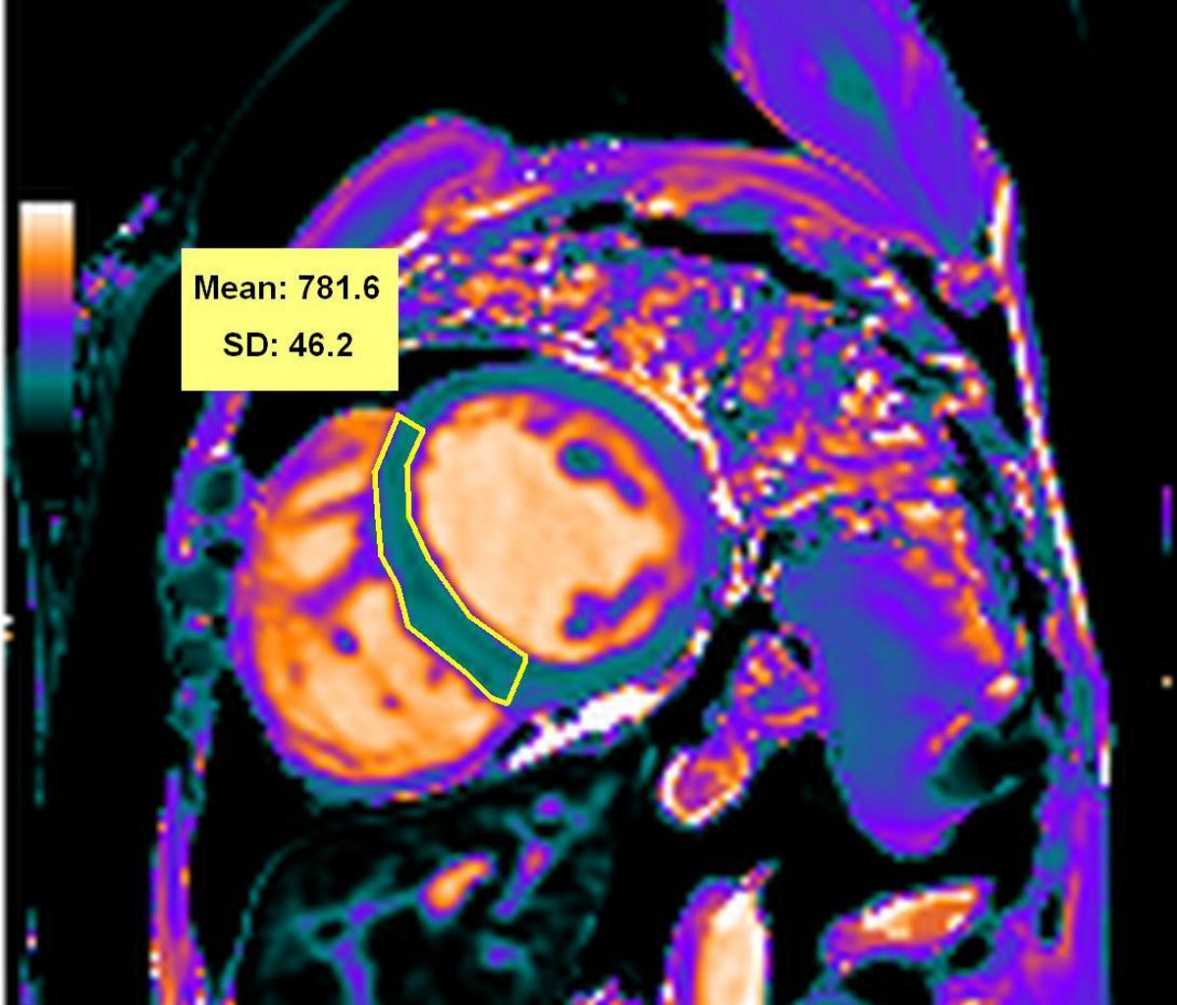
T1 map at 3T of a patient with iron overload showing reduced T1 time within the interventricular septum (781.6 ms), in agreement with a significantly reduced T2* time at 1.5T (8.5 ms - not shown).

A total of 15 normal volunteers and 7 chronic anemia patients (with a myocardial T2* measure <20 ms at 1.5T in the last 2 years, five of these on iron chelating therapy) were prospectively enrolled. Myocardial T2* and T1 times were quantified in the same day, the former using a breath-hold multi-echo GRE sequence at 1.5T (Symphony, Siemens, Erlangen, Germany) and the latter using the T1 mapping -MOLLI sequence at 3T (Verio, Siemens, Erlangen, Germany). All ROIs were placed at mid-interventricular septum, carefully avoiding the blood pool (Fig 1). All analyses were blinded.

## Results

**Figure 2:**
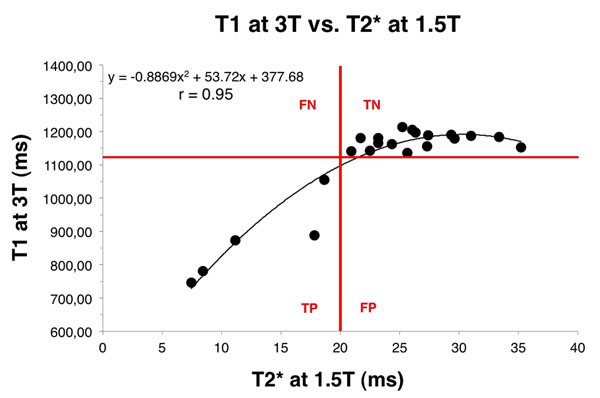
Correlation curve between T1 at 3T and T2* at 1.5T. The whole data were best fitted by a quadratic curve with r=0.95. Red lines delimitate true positives (TP), true negatives (TN), false positives (FP) and false negatives (FN) based on a T1 cutpoint of 1130 ms for the prediction of a T2* < 20 ms.

All patients had regular heart rhythm and all MRI exams showed diagnostic image quality. Volunteers and patients had significantly different mean myocardial T2* (27.2 ms +/- 3.9 vs. 15.4 ms +/- 6.3 p<0.05 respectively) and T1 times 1175.7 ms +/- 22.8 vs. 952.1 ms +/- 173.2 p<0.05 respectively). 3T T1 times strongly correlated with 1.5T T2* times (r=0.95 and Fig 2). Using the 3T T1 cut-off of 1130 ms, sensitivity and specificity for 3T T1 to predict a T2*<20 ms at 1.5T (standard reference) were both 100%.

## Conclusions

Myocardial T1 value obtained with a MOLLI sequence has excellent iron quantification capability at 3T.

## Funding

Internal.

